# Language skills differences between adults without formal education and low formal education

**DOI:** 10.1186/s41155-021-00205-9

**Published:** 2022-01-04

**Authors:** Ariane Pereira, Karin Zazo Ortiz

**Affiliations:** Department of Speech, Language and Hearing Sciences, Universidade Federal de São Paulo (UNIFESP), Rua Botucatu, São Paulo, 802 Brazil

**Keywords:** Language, Educational status, Neuropsychological tests, Language disorders, Aphasia

## Abstract

**Background:**

The influence of education on cognition has been extensively researched, particularly in countries with high levels of illiteracy. However, the impact of low education in all cognitive functions appears to differ. Regarding to language, the effects of education on many linguistic tasks—supported by different processing—remain unclear. The primary objective of this study was to determine whether oral language task performance differs among individuals with no formal and low-educated subjects, as measured by the Brazilian Montreal-Toulouse Language Assessment Battery (MTL-BR). This is the only language battery available for use in Brazil, but lacks normative data for illiterate individuals. The secondary objective was to gather data for use as clinical parameters in assessing persons with aphasia (PWA) not exposed to a formal education.

**Methods:**

A total of 30 healthy illiterate individuals aged 34–60 years were assessed. All participants underwent the MTL-BR Battery, excluding its written communication tasks. The data obtained in the present study were compared against results of a previous investigation of individuals with 1–4 years of education evaluated using the same MTL-BR instrument.

**Results:**

Statistically significant differences in performance were found between non-formal education and the low-educated (2–4 years) groups on the tasks Auditory Comprehension, Repetition, Orthographic/Phonological Fluency, Number dictation, Reading of numbers and also on simple numerical calculations.

**Conclusion:**

The study results showed that individuals with no formal education/illiterate had worse performance than low-education individuals on some of the language tasks of the MTL-Br Battery, suggesting that each year of education impacts cognitive-language performance. Also, data were obtained which can serve as a guide for PWA not exposed to a formal education.

## Introduction

Illiteracy is a global problem given that many countries, particularly developing nations, still have high rates of illiteracy. According to data from the Brazilian Institute of Geography and Statistics [IBGE] ( n.d.), in 2015, the illiteracy rate in Brazil among individuals aged ≥ 15 years was 8%. Most of people that are illiterate have never been exposed to a formal education. Considering the frequency of illiteracy in the world, researches have studied the influence of lack of schooling on cognitive functions (Colaço et al., 2010; Noronha et al., 2018; Teruya et al., 2009).

With regard to language, many previous studies (Ortiz et al., 2006; Ortiz & Costa, 2011; Radanovic et al., 2004) involving language assessment have confirmed that education influences linguistic performance. Education not only influences written tasks (Radanovic et al., 2004; Soares & Ortiz, 2009) and more complex tasks (Zanichelli et al., 2020) as might be expected, but also impacts oral-based tasks (Mansur et al., 2005), such as comprehension of complex sentences (Ortiz & Costa, 2011; Ortiz et al., 2006), repetition of pseudo-words and phrases (Ortiz et al., 2006) and verbal fluency (Meguro et al., 2001).

A major language disorder that can affect adults with brain damage is aphasia. Stroke is the main cause of aphasia today (Dickey et al., 2010; National Aphasia Association, n.d.). Aphasia is present in 21–38% of acute stroke patients and is associated with high short- and long-term morbidity, mortality, and expenditure (Berthier, 2005). Unfortunately, although mortality rates for stroke have decreased, the absolute number of people who have stroke annually is increasing, with most of the burden in low- and middle-income countries (Krishnamurthi et al., 2013), such as Brazil.

In view of the consensus that education impacts cognitive functions, it is important that language assessment be based on reliable parameters for this group. It is also relevant given the formal nature of the tests applied, which may promote anxiety over the novel experience of testing. Besides, low education can simulate or overshadow the effects of neurological damage (Beausoleil, Fortin, Le Blanc, and Joanette, 2003) leading to false positive results (Akashi & Ortiz, 2018; Nunes et al., 2009).

Regarding the assessment of persons with aphasia (PWA), formal assessment is especially important for the longitudinal follow-up and for the objective control of the gains obtained with rehabilitation. In Brazil, one validated is the MTL -BR Battery. The normative data presented, however, have been obtained from adults with at least 5 years of formal education. More recently, a pilot study (Akashi & Ortiz, 2018) applying the MTL-BR revealed differences in performance between subjects with 5–8 years’ education and 2–4 years of formal education. The disparities found were so marked that, on some linguistic tasks, individuals in the less education group were diagnosed with language disorders. This finding suggests that, every year of formal education, particularly in the early stages of learning, can modify and impact cognitive performance. If it is specifically true, it is expected that people no formal education perform even worse on the MT-BR tasks. Besides, by the other hand, would be helpful to investigate which tasks low-educated or individuals with no formal education could perform as well as high-educated individuals. Then, based on the hypothesis that each year of education contributes to cognitive development, the primary objective of the present study was to compare the performance of individuals with no formal education versus low-educated subjects on the language subtests from the MTL-BR Battery to determine the effect of formal education on language. If a clinical difference is found (as expected), the data obtained from this study can be observed and considered when PWA with no previous formal education are exposed to the test and also could highlight specific tasks that probably not suffer the interference from a formal education.

## Methods

The study was approved by the Research Ethics Committee of the Universidade Federal de São Paulo (permit no. CEP0036/2018) and conducted at the Neuropsycholinguistics Laboratory of the Department of Speech, Language and Hearing Sciences of the Universidade Federal de São Paulo. All participants first signed the informed consent form, devised according to the recommendations of the National Health Council in compliance with Resolution no. 466/12, thereby agreeing to take part in the study on a voluntary basis. The form was read out aloud in full to each participant by a witness together with the researcher. The witness signed all pages of the form, which were also signed, initialed or marked by finger print by the participants.

### Participants

Thirty adults with no formal education and illiterate were assessed. The participants were students enrolled to join the Youth and Adult Education Program (Educação de Jovens e Adultos-EJA). This is a program to adult literacy. Adults that were enrolled but had not yet started the literacy program were invited to participate in the study. Then, the data collected in the study were compared against those of a previous study (permit no. CEP 1219/2017) (Akashi & Ortiz, 2018) with 30 individuals with education ranged from 2 to 4 years.

Participants from both studies were selected based on the following inclusion criteria: age 18–60 years and normal performance on the cognitive screening tests. Study exclusion criteria for both groups were history of previous or current language impairments, diagnosis or history of visual or auditory deficits which could prevent execution of the test, history of current or previous psychiatric or neurological disorders, and use of any legal or illegal psychotropic drugs (except atypical neuroleptics) or alcohol abuse.

### Procedures

The information about the inclusion criteria was collected by applying the Questionnaire on Health Conditions and Sociocultural Aspects.

The Minimental State Examination (MMSE) was used as a screening tool (Folstein, Folstein, and Mchugh, 1975). We adopted a Portuguese-translated version, with cut-off scores adapted to the subjects’ educational levels (Brucki et al., 2003): illiterate = 20; elementary (1 to 4 years of education) = 25; 5 to 8 years of education = 26.5; 9 to 11 years of education. This test was chosen because it was considered a good procedure for cognitive screening even with low education levels (Castro-Costa et al., 2009). Those individuals whose performance was normal on the cognitive screening test were submitted to the Language Assessment Battery (MTL-BR). The battery was applied according to the guidelines described in the instrument (Parente et al., 2016). Given that subjects to be tested had no formal education, the tasks assessing written communication (written comprehension, copying, written dictation, reading aloud, written naming, written narrative discourse, and written text comprehension) of the MTL-BR Battery were excluded. All of the volunteers were assessed individually by the same examiner in a quiet room, with assessments taking an average of 1 h to complete. All procedures were done at the same day. Only the MTL-BR tasks outlined below were performed:

1. Directed interview: includes 13 open-ended questions to analyze speech and auditory comprehension. The total score is 26 points: 13 items with maximum score of two points each.
2. Automatic speech: assesses the ability to evoke automatisms such as numbers, days of the week and the birthday song. Total score ranges from 0 to 6 points.
3. Auditory comprehension: measures the ability to identify images that represent words and phrases from auditory input. The task consists of a total of 19 items, five words (boards with six stimuli comprising one target and five distracters: one phonological, one semantic, one visual and two neutral) and 14 sentences .The maximum score is five points for words and 14 points for phrases, with one point for each correct answer.
4. Oral narrative task: Evaluates the ability to tell a story from visual inputs. The task consists of describing a picture depicting a bank robbery. The narrative was analyzed for the number of words produced.
5. Repetition: The task consists of 11 words (8 words and 3 non-words) and three sentences. The maximum scores are 11 and 22 points for words and phrases respectively, with one point for each word produced correctly, yielding a maximum score of a 33 points.
6. Semantic verbal fluency: Evaluates spontaneous production of words in the category “animals” within a time period of 90 s. Each word correctly selected from this class is equivalent to one point, ignoring repetitions, morphological derivatives of the same word, and other words that do not match the requested category.
7. Non-verbal praxis: assesses the ability to produce isolated gestures and movement sequences involving the face and tongue, requested by the evaluator through verbal instructions. The task consists of a total of 6 items with maximum scores of 4 points each, giving a maximum total of 24 points.
8. Naming: measures the ability to identify and name pictures that refer to nouns and verbs, from a visual input. Fifteen pictures are presented (12 nouns and three actions), placed on individual boards. The maximum score is 30 points, comprising 15 items with a maximum score of two points each. The criteria for scoring is incorrect answer: zero; item semantic related or description: 1 point; and correct answer: 2 points.
9. Object manipulation: Assesses the ability to understand simple and complex commands. The individual is instructed to perform six commands given by the clinician, using physical objects (key, comb, cup, pen, and paper). The complexity of orders increases gradually. The maximum score is 16 points. One point is given to each part of the command that is properly performed by the individual.
10. Phonological verbal fluency: evaluates spontaneous production of words that start with the letter M within a time period of 90 s. For all participants, when it was a necessary, it was also given a sound/phoneme clue /m/. Each correct word equals one point, ignoring repetitions, morphological derivatives of the same word and proper names.
11. Body part recognition and left-right orientation: assesses the individual's ability to identify parts of the body and their laterality. The maximum score is eight points, of which four points are given for each body part (limbs) and the other four are given for the right-left orientation.
12. Oral text comprehension: assesses the ability to understand auditory input from a text read by the clinician. The individual must answer six questions orally after listening to the text (three open-ended and three closed-ended questions). The maximum score is nine points: a maximum of two points for each of the three open-ended questions (for instance, what was stolen?) and one point for each of the closed-ended questions (Did the thief want to kidnap the baby?).
13. Number dictation: Assesses the ability to understand the auditory stimulus and write down the corresponding number in Arabic form. The task consists of six numbers. Each number written correctly gets one point, with a maximum score of six points.
14. Reading of numbers: assesses the ability to recognize Arabic numerical and visual stimuli and reproduce them orally. The maximum score is six points: one point for each number read correctly.
15. Calculation: evaluates the ability to perform the numerical operations of addition, subtraction, multiplication and division, as well as mental simple mathematical problem. The maximum score is 6 points.

The tasks dictation of Arabic number, reading of Arabic numbers and calculation were remain in this study because a previous study (De Luccia & Ortiz, 2009) observed that some mathematic tasks can performed by individuals with low education probably because number processing are very common in daily activities. As stimulus from MTL-BR vary in complexity (i.e., 8 and 6024) and also have simple calculus (for instance 5 + 2), in this study, we wanted to investigate how individuals with no formal education could perform these tasks.

Verbal fluency tasks and oral narrative discourse tasks were scored according to number of words produced, whereas the remaining tasks were scored according to number of correct answers.

### Statistical analysis

The data obtained were submitted to statistical treatment. Mean, median, standard-deviations, maximum, and minimum limits were established to serve as reference parameters for all groups on the tasks evaluated.

Analysis of the comparison of the study groups (low-educated versus non formal education) for performance on the tasks from the MTL-BR battery, was carried out using multivariate covariance analysis (MANCOVA) using Pillai’s multivariate screening test. First, we noted that the age of the non-formal education group (mean = 49.13 years, SD = 7.87 years) was significantly older than the low-educated group (mean = 44.30 years, SD = 8.75 years). Then, age was incorporated as a covariable in the general linear model constructed for this analysis to control for its effect, given that mean age of the two groups differed significantly (Student’s *t* test, *p* = 0.028).

A statistically significant difference was also found between the groups on the MTL-BR tasks performed (*p* < 0.001)—a difference which persisted after controlling for the effect of age (*p* < 0.001). To investigate which tasks differed for performance between the groups, univariate analysis of covariance (ANCOVA) was carried out for each variable, with Bonferroni correction for multiple comparisons. Age was again incorporated into the general linear model built for this set of analyses to control for the effect of the variable, given the mean age of the groups differed significantly.

The value for statistical significance adopted was 5% (*p* = 0.05).

## Results

The performance of healthy low-educated individuals was compared with that of healthy individuals with no formal education on the tasks from the MTL-BR battery. Statistically significant differences were found between the groups on some MTL-BR subtests, where low-educated individuals had better performance than individuals with no formal education. The differences in performance between the two groups are shown in Table 1. They were auditory comprehension, oral narrative discourse, repetition, phonological/orthographic verbal fluency, number dictation, reading of numbers, and numerical calculation.

**Table 1:** MTL-BR tasks in which statically differences where found between NFE and LE groups

MTL-BR	Group	Mean	Standard deviation	Median	Minimum	Maximum	*p*^a^
Auditory comprehension	NFE	14.93	1.87	16.00	11.00	18.00	.007*
	LE	16.80	1.35	17.00	14.00	19.00	
Oral narrative discourse	NFE	81.40	44.05	77.50	26.00	192.00	.001*
	LE	40.73	17.42	35.50	14.00	87.00	
Repetition	NFE	31.27	1.64	32.00	25.00	33.00	.001*
	LE	32.73	0.69	33.00	30.00	33.00	
Phonological/orthographic verbal fluency	NFE	4.37	4.05	3.50	0.00	16.00	< .001*
	LE	9.80	4.34	10.00	2.00	21.00	
Number dictation	NFE	3.97	1.35	4.00	1.00	6.00	.010*
	LE	5.10	0.71	5.00	4.00	6.00	
Reading of numbers	NFE	4.23	1.01	4.00	2.00	6.00	< .001*
	LE	5.20	0.61	5.00	4.00	6.00	
Numerical calculation	NFE	3.00	1.36	3.00	0.00	7.00	< .001*
	LE	5.83	3.22	6.00	0.00	12.00	

*Note*: *NFE* non-formal education group, *LE* low-educated group

^a^*p* value controlling for effect of “age” factor*Statistically significant value at 5% level (*p* ≤ .05)

Table 2 shows the performance from the both groups of individuals on tasks that do not seem to suffer the interference of education. Mean, *SD*, median, minimum, and maximum values obtained in both groups are shown.

**Table 2:** Mean, SD, median, minimum, and maximum values obtained in MTL-BR tasks according to study group. Tasks with no statistical difference observed

MTL-BR	Group	Mean	Standard deviation	Median	Minimum	Maximum	*p*^a^
Structured interview	NFE	25.07	0.94	25.00	23.00	26.00	> .999
	LE	25.37	1.03	26.00	21.00	26.00	
Automatic speech	NFE	5.20	0.71	5.00	4.00	6.00	.109
	LE	5.73	0.58	6.00	4.00	6.00	
Semantic verbal fluency	NFE	15.93	4.49	16.00	7.00	26.00	> .999
	LE	16.47	4.22	16.00	7.00	25.00	
Nonverbal praxis	NFE	23.13	1.17	24.00	20.00	24.00	> .999
	LE	23.60	0.97	24.00	20.00	24.00	
Naming	NFE	25.60	2.28	26.00	21.00	29.00	> .999
	LE	27.00	3.40	28.00	15.00	30.00	
Object manipulation	NFE	15.53	0.86	16.00	13.00	16.00	.454
	LE	15.97	0.18	16.00	15.00	16.00	
Body part recognition	NFE	8.00	0.00	8.00	8.00	8.00	> .999
	LE	7.87	0.73	8.00	4.00	8.00	
Oral comprehension: text	NFE	4.73	1.70	5.00	1.00	8.00	> .999
	LE	5.53	2.52	6.00	0.00	9.00	
Mental numerical calculation	NFE	2.77	1.01	3.00	0.00	4.00	> .999
	LE	3.03	1.56	3.00	0.00	6.00	

*Note*: *NFE* non-formal education group, *LE* low-educated group

^a^*p* value controlling for effect of “age” factor *Statistically significant value at 5% level (*p* ≤ .05)

## Discussion

The results of the present study revealed worse performance by individuals with no formal education than low-educated subjects on several oral language tasks from the MTL-BR Battery. This study corroborated the findings of previous reports in the literature (Radanovic, Mansur, and Scaff, 2004; Soares & Ortiz, 2009) showing that education influences the performance of healthy individuals on language tasks, namely: oral comprehension, oral narrative discourse, word repetition, phonological/orthographic verbal fluency, number dictation, number reading, and written numerical calculations (Table 1). Individuals with no formal education had worse performance than low-educated subjects.

In a previous study, Akashi and Ortiz (2018) investigated the influence of low education on the language tasks assessed by the MTL-BR Battery and highlighted the importance of developing more studies with larger populations, including more individuals with 1 and 2 years of formal education and with no formal education, since there are still no normative data available for these populations for the MTL-BR Battery use. Their results lend support to the hypothesis that even a few years of education affects language performance and hence impacts scores on the different MTL-BR subtests. In this study, statistically significant differences in performance by low-educated individuals relative to individuals with no formal education were observed and a minimum level of education appears to change cognitive performance. These findings will be discussed below.

There was a difference between the performance of NFE and LE groups in the auditory comprehension subtest. This task measures the ability to identify images that represent words and phrases from auditory input. However, the qualitative analysis showed better performance on oral comprehension of words than of sentences for both groups, with several possible explanations for this disparity. Pictures depicting actions require a larger number of visual inferences than single items, while the core components for understanding an image are inherently more detailed, which in turn requires correct visual perception of both agent and object (Mansur et al., 2005). In addition, greater demand is placed on working memory to process sentences with non-canonic structure, such as those with passive structures and center-embedded clauses, since the phrase must be first stored, then organized and syntactically decoded, for final comprehension of the information and selection of the correct drawing (Eom & Sung, 2016; Ortiz & Bertolucci, 2005). Previous studies have found differences in oral comprehension of sentences when groups with 1–4 years of formal education and 5–8 years of formal education were compared (Akashi & Ortiz, 2018) and even in comparison of 5–8 years of education to 8–12 years of education (Pagliarin et al., 2014) and, in this case, it can also be influenced by reading and writing habits (Pagliarin et al., 2015). Finally, the oral comprehension of text requires comprehension, retention and retrieval of the information presented in the text, recruiting working memory and components of executive functions. However, working memory and metacognitive operations are mediated and boosted by formal education (Ardila, Rosselli, and Rosas, 1989; Jou & Sperb, 2006), possibly explaining the lower performance by individuals with no formal education compared to low-educated individuals on this task.

Regarding to the oral language tasks investigated in this study, the participants presented low performance on oral narrative discourse task. The narrative comprises a description of a series of events and actions. The manner in which individuals explain the actions of the characters resembles the way in which they construe actions of people in everyday life. Therefore, for this process to function effectively, inferences must be created (Bower & Morrow, 1990). These inferences can be explained as mental representation formed through interaction between explicit linguistic information and world knowledge held by the individual (Alonso, 2004; Zanichelli, Fonseca, and Ortiz, 2020). These inferences aid the construction and comprehension of discourse (Alonso, 2004) and depend on the world knowledge held by the individual and might explain why, during this test, the discourse produced tended to be based on the individual´s personal experiences of the world/everyday life when the meaning of the image could not be grasped, resulting in stories unrelated to the theme presented. The individuals may have encountered difficulty understanding the context of the target situation and/or problems accessing their scripts (a bank robbery—in the case of the MTL-BR) on world knowledge or use them properly to make pragmatic judgments correctly (Hirst, LeDoux, and Stein, 1984). It is likely that, due to a break down in the inferential process, the individuals produced descriptive discourse. The failed integration of the elements present in the stimulus must have led the subjects to describe each object in the drawing in detail, without correlating them to form a story. The main components of a target figure are directly related to the generating of inferences (Ribeiro & Radanovic, 2014). Thus, if the individuals had fewer information units regarding the scene from the outset, they likely made fewer visual inferences, explaining the poorer narrative discourse produced. In fact, successful discourse requires the combination of information units, as propositions, in a coherent way to convey a significant message (Wright, 2011).

On the repetition task, the individuals with no formal education had major difficulty and presented lexicalization errors, suggesting the use of the lexical route when the phonological route was needed. The phonological route starts, naturally, by a phonological analysis of the auditory input. A phonological input buffer permits the storage of phonological information (segmented and correctly sequenced) for a short period (Reis & Castro-Caldas, 1997). The repetition of non-words demands comparisons or the detection of the specific phonemic characteristics of words. It seems that it is precisely this analysis that is problematic in illiterate individuals, who demonstrate difficulties in certain tasks that required phonological awareness (Reis & Castro-Caldas, 1997). In fact, the MTL-BR Battery has pseudo-words that depends on the phonological route responsible for phonemic coding typically underdeveloped in illiterate or low-educated subjects (Petersson, Reis, Askelöf, Castro-Caldas, and Ingvar, 2000).

The individuals with no formal education also had worse results on Arabic number dictation and Arabic number reading, as well as on mental and written mathematics calculations. First, the option to investigate the performance of individuals with no formal education on these tasks is because numbers are present and mathematical calculation exists in many everyday activities. Calculation ability under normal circumstances requires not only the comprehension of numerical concepts, but also that of conceptual abilities and other cognitive skills, so it is impossible predict the exact impact of daily activities on number learning and processing. The difficulties on Arabic number reading and Arabic number dictation were more marked for numbers containing hundreds and thousands. These findings corroborate previously results (De Luccia & Ortiz, 2009) showing that low education impacted performance on some mathematics tasks, such as orthographic transcoding of numbers. On mental mathematical calculations, the subjects had no problems for addition or subtraction, but all encountered difficulties with multiplication and division. This is particularly true for carrying out multiplications, which need knowledge of the times table, the most commonly used approach for teaching multiplication in Brazil. It is worth mentioning that individuals with no formal education performed mental calculation as well as with low-educated individuals (Table 2). This pattern probably occurred because simple addition and subtraction are more commonly used in everyday situations than other operations requiring more formal learning. This finding is important in as far as it supports the notion that, although analyzing the effect of education on individual performance during neuropsychological tests is paramount, the influence of social environment should also be investigated. This environment dictates whether the individual received stimuli to develop certain abilities or otherwise, further contributing to cognitive performance. For written mathematical calculations, individuals with no formal education were unable to solve, irrespective of mathematical operation involved (addition, subtraction, division, or multiplication), possibly because at this part of the task, the mathematical operations are more complex and then, probably more dependent of learning obtained through a formal education. Indeed, level of familiarity with carrying out arithmetic increases with years of formal education (De Luccia & Ortiz, 2009).

On the semantic verbal fluency task, participants had difficulty producing words from the animal’s category and, on numerous occasions, participants were in doubt over whether a given word belonged to the category. This difficulty might be explained by the fact that formal education facilitates the organizing of semantic subgroups and categories (Ratcliff, Ganguli, Chandra, Sharma, Belle, Seaberg, and Pandav, 1998). Nevertheless, no statistically significant difference between no formal education and low-educated groups was evident. Considering this task, comparing the data from this study with data from healthy individuals with 5–8 years of education, differences were found and they are possibly owing to sociocultural influence and learning through lifespan. Animals are very familiar category. As outlined earlier, although education is a determinant of cognitive performance, it is not the only variable to consider. The role of stimulation and probable influence of the sociocultural environment on cognitive development should also be taken into account. These aspects, however, are difficult to measure objectively.

For phonological verbal fluency, the subjects exhibited great difficulty producing words, most likely explained by the connection between development of phonological abilities and formal education. Most studies investigating the impact of literacy on oral language processing have shown that literacy provides phonological awareness skills in the processing of oral language and the ability to segment speech into phonemic units is dependent on literacy (Tsegaye, De Bleser, and Iribarren, 2011; Ratcliff, Ganguli, Chandra, Sharma, Belle, Seaberg, and Pandav, 1998). Our results suggest the existence of differences in phonological processing even between individuals with no formal and low formal education, as was demonstrated by Ardila, Ostrosky, and Mendoza (2000) and Colaço, Mineiro, Leal, and Castro-Caldas (2010).

Finally, on the other hand, Table 2 shows tasks from MTL-BR Battery that no differences were found between individuals with no formal education and low-educated ones. Since MTL-BR Battery was published, several studies investigated scores in large populations, considering the variables age, years of schooling (Akashi & Ortiz, 2018; Pagliarin et al., 2014) and other sociocultural variables such as reading and writing habits (Pagliarin et al., 2015). Taken together the data from these previous studies and the present study, it can be observed that the tasks structure interview, automatic speech (content), nonverbal praxis, object manipulation and body part recognition seem not be influenced by years of schooling. The structure interview is a task that is based on a series of questions, most of them about people’s daily lives. It is a conversational task, and probably the questions can be easily understood and answered by people, regardless of sociodemographic variables. In the case of the object manipulation task, auditory, proprioceptive and visual processing are involved in its execution. The familiarity of the objects presented and the tangible effect they evoke may have also facilitated execution of the task. These factors likely promoted the similar performance in carrying out the task (Medeiros & Ortiz, 2021). In addition, results of a previous study (Akashi & Ortiz, 2018) revealed the presence of a ceiling effect on this task among healthy individuals with low educational level. In turn, the absence of differences between groups in automatic speech and non-verbal praxis tasks were expected. Automatic series are considered the linguistically simpler tasks and non-verbal praxis only requires individuals to perform movements involving the tongue and lips. So, even considering more ecological or informal methods to assess PWA with no formal or low education, these tasks can be used and probably can be helpful to identify language and speech disorders and follow-up of these patients.

This study has clinical applicability because the MTL–BR is the only language assessment battery for adults available in Brazil, rendering it especially important to determine the effects of education on task performance. Although a pilot study, the data gathered can be a guide during the application of the MTL-BR Battery in PWA with no formal education.

### Study limitations

This was a pilot study investigating the influence of no formal education on performance on language tasks measured by the instrument. However, studies involving larger populations and studies that control possible influence of the socioeconomic status are needed to confirm these findings with individuals with no formal education on the MTL-BR Battery. The significant differences in performance between no formal education and low-educated groups on the language tasks assessed strongly suggest that populations with little formal education should be studied in greater depth, perhaps according to each year of education as opposed to the broader education bands (1–4 years) used in most studies.

## Conclusion

The study results showed that individuals with no formal education had worse performance on some of the language tasks of the MTL-BR Battery relative to healthy low-educated subjects and seems to confirm the hypothesis that each year of education contributes to cognitive development. Moreover, data was gathered on this group that can serve as clinical guide for assessing PWA with no formal education.

## Data Availability

The datasets used and/or analyzed during the current study are available from the corresponding author on reasonable request.

## Abbreviations

IBGE: Brazilian Institute of Geography and Statistics
MTL-BR: Brazilian version of Montreal Toulouse Language Assessment Battery
EJA/YAE: Educação de Jovens e Adultos/Youth and Adult Education
MMSE: Mini-Mental State Exam
